# Water as a Drink

**Published:** 1881-05

**Authors:** 


### WATER AS A DRINK.

MANY persons drink ordinarily as little water as possible, and none at all at meal times, because they suppose that water dilutes the gastric juice. Experiments, however, show that dilution does not diminish the digestive power of the gastric juice, and further, that water alone, as well as solid food, awakens its secretion.

A paper, read by Dr. Webber, of Boston, at a meeting of a learned medical society, took the ground that water, used moderately at meals, is beneficial; and that a large class of persons drink too little.

The result is, if too little water is drunk—especially if the person eats heartily—the perspiration and the kidney secretions are diminised. Not only they, but the waste of the system which can be removed only in a state of solution, is not eliminated with sufficient regularity and fullness, and the system becomes gradually clogged by it. The accumulation is slight from day to day, but in time unpleasant symptoms are developed.

These symptoms are of an indefinite character—discomfort, even pain, sometimes in one place and sometimes in another, constipation, and unhealthy hue of the skin.

"Patients," said Dr. Webber, "who drank no more than a pint of water a day, have told me that they were not thirsty. They were surprised when told to drink more. Those who have followed this suggestion in the course of a week have developed thirst, and drank as many as three pints of water a day."

We may add that water taken into the stomach is at once rapidly absorbed by the blood vessels. A bowl of well-seasoned broth as a first course is specially helpful to the above class of patients. A large quantity of ice-cold water is harmful to any one.—*Youth's Companion.*

### SELF-SUSTAINING.

The simplest post-office in the world is in Magellan Straits, and has been established there for some years past. It consists of a small cask, which is chained to the rock of the extreme cape in the straits, opposite Terra del Fuego. Each passing ship sends a boat to open the cask and to take letters out and place others in it. The post-office is self-acting therefore ; it is under the navies of all nations, and up to the present there is not one case to report n which any abuse of the privileges it affords has taken place.

